# Transcutaneous monitoring of hemoglobin derivatives during methemoglobinemia in rats using spectral diffuse reflectance

**DOI:** 10.1117/1.JBO.26.3.033708

**Published:** 2021-02-13

**Authors:** Fahima Khatun, Yoshihisa Aizu, Izumi Nishidate

**Affiliations:** a Tokyo University of Agriculture and Technology, Graduate School of Bio-Applications and Systems Engineering, Tokyo, Japan; b Bangabandhu Sheikh Mujibur Rahman Agricultural University, Faculty of Veterinary Medicine and Animal Science, Department of Pathobiology, Gazipur, Dhaka, Bangladesh; c Muroran Institute of Technology, College of Design and Manufacturing Technology, Hokkaido, Japan

**Keywords:** hemoglobin, methemoglobinemia, spectral imaging, multiple regression analysis, Monte Carlo simulation

## Abstract

**Significance:** Untreated methemoglobinemia may cause severe hypoxemia and even death when methemoglobin levels in the blood stream exceed 70%. Although CO-oximetry can be used to monitor the response to treatment for methemoglobinemia, it is costly and requires an invasive procedure for collecting blood samples from patients. A pulse CO-oximeter with a contact probe can be used to continuously and non-invasively measure the percentage of methemoglobin, as well as the percutaneous oxygen saturation. In terms of the prevention of infectious diseases, however, it is desirable to monitor methemoglobin and oxygen saturation levels in a non-contact manner. Diffuse reflectance spectral imaging is promising as a non-contact, non-invasive, and cost-effective clinical diagnostic tool for methemoglobinemia.

**Aim:** To demonstrate the feasibility of visible spectral diffuse reflectance for *in vivo* monitoring of hemoglobin derivatives and evaluating methemoglobin production and reduction as well as hypoxemia during methemoglobinemia in rats.

**Approach:** A new imaging approach based on the multiple regression analysis aided by Monte Carlo simulations for light transport was developed to quantify methemoglobin, oxygenated hemoglobin, and deoxygenated hemoglobin using a hyperspectral imaging system. An *in vivo* experiment with rats exposed to sodium nitrite (${\mathrm{NaNO}}_{2}$) at different doses was performed to confirm the feasibility of the method for evaluating the dynamics of methemoglobin, oxygenated hemoglobin, and deoxygenated hemoglobin during methemoglobinemia. Systemic physiological parameters, including the percutaneous arterial oxygen saturation, heart rate (HR), and pulse distention, were measured by a commercially available pulse oximeter, and the results were compared to those obtained by the proposed method.

**Results:** Both the methemoglobin concentration and methemoglobin saturation rapidly increased with a half-maximum time of <20  min. They reached their maximal values nearly 60 min after the administration of ${\mathrm{NaNO}}_{2}$. Tissue oxygen saturation dramatically dropped to a minimum of 33.7%±0.4%, 23.1%±5.6%, 8.8%±1.7%, and 9.7%±5.1% on average for ${\mathrm{NaNO}}_{2}$ doses of 25, 37.5, 50, and 75 mg/kg, respectively. Changes in methemoglobin concentration and tissue oxygen saturation are indicative of the temporary production of methemoglobin and severe hypoxemia during methemoglobinemia. Profound increases in the HR and pulse distention implied an elevated cardiac output caused by tachycardia and the resultant increase in peripheral blood volume to compensate for the hypoxia and hypoxemia during methemoglobinemia. This was in agreement with the time course of the peripheral hemoglobin volume concentration obtained by the proposed method.

**Conclusions:** The proposed method is capable of the *in vivo* non-contact simultaneous evaluation of methemoglobin levels and hypoxemia during methemoglobinemia, and that it has potential as a tool for the diagnosis and management of methemoglobinemia.

## 1 Introduction

**Fig. 1:**
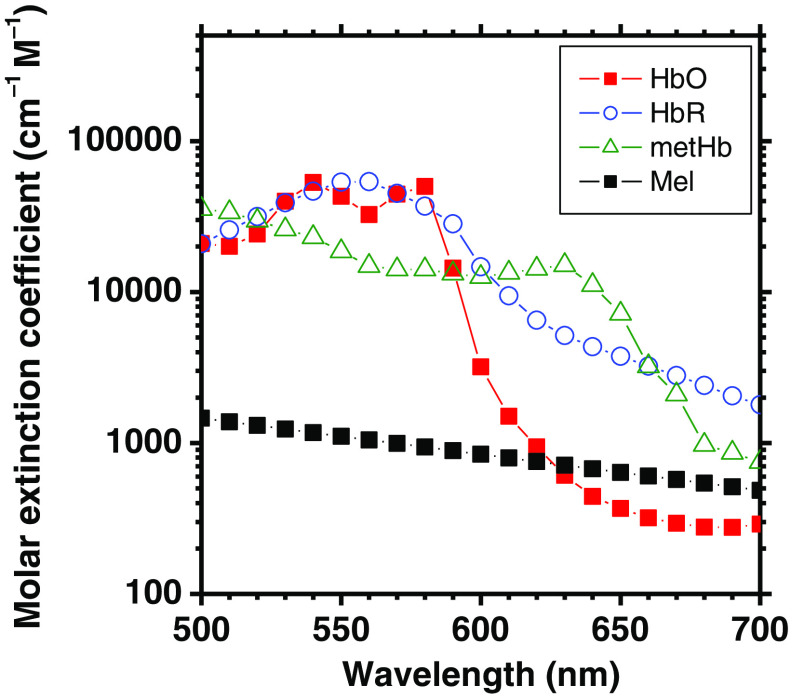
Extinction coefficient spectra of typical hemoglobin derivatives (oxygenated hemoglobin HbO,^2^ deoxygenated hemoglobin HbR,^2^ and methemoglobin^3^) and melanin^4^ in the visible wavelengths between 500 and 700 nm.

The spectral features of human skin are often affected by various skin diseases, which cause altered attenuation of light within multiple layers of the skin. In the epidermis and dermis, similar changes are associated with fluctuations in the concentration of melanin and blood-borne pigments, such as hemoglobin.^1^ Each chromophore has its own characteristic absorption spectrum in the visible to near-infrared wavelength regions, which change with wavelength and can function as a fingerprint for that molecule. The binding of other molecules to the functional chromophore causes variations in the structure of a molecule, resulting in an altered absorption spectrum for each derivative of a molecule that is formed. Figure 1 shows the extinction coefficient spectra of typical hemoglobin derivatives and melanin at visible wavelengths between 500 and 700 nm.^2^^–^^4^ The actual absorption spectra of hemoglobin depend primarily upon whether or not the blood is oxygenated, and there are thus two main derivatives, i.e., oxyhemoglobin in oxygenate blood and deoxyhemoglobin in deoxygenated blood. There are also hemoglobin derivatives that are incapable of transporting oxygen molecules, and they are, therefore, collectively known as dyshemoglobins; these include methemoglobin, carboxyhemoglobin, and sulfhemoglobin. The absorption can differ drastically between dysfunctional hemoglobin and functional hemoglobin.^5^^,^^6^

Methemoglobin is a dysfunctional form of hemoglobin, in which the ferrous (${\mathrm{Fe}}^{2+}$) irons of heme are oxidized to the ferric (${\mathrm{Fe}}^{3+}$) state and cannot bind oxygen molecules. Therefore, methemoglobin is incapable of transporting oxygen molecules to peripheral tissues.^7^ As a consequence, a large amount of methemoglobin causes methemoglobinemia, which leads to tissue hypoxia.^8^ Under normal physiological states, only a small amount (<2%) of hemoglobin exists in the form of methemoglobin.^6^ However, a higher percentage is found in individuals who suffer from methemoglobinemia due to genetic diseases^9^^–^^12^ or exposure to certain chemicals and drugs.^13^^–^^17^ Severe untreated methemoglobinemia is life-threatening and may cause death when methemoglobin levels in the blood stream exceed 70%.^6^^,^^18^ There are many case reports of potentially serious methemoglobin levels induced by topical anesthesia with lidocaine and benzocaine.^15^^,^^16^ As with other potentially life-threatening sicknesses, early assessment of methemoglobinemia is essential for effective monitoring and treatment.

The conventional methods for evaluating methemoglobin in hypoxemic patients are arterial blood gas analysis, pulse oximetry, and CO-oximetry. Arterial blood gas analysis results could show a normal oxygen partial pressure (${\mathrm{pO}}_{2}$) even when there is a high concentration of methemoglobin in the blood. This is because arterial blood gas analysis calculates only the dissolved oxygen and not the actual amount of oxygen molecules bound to hemoglobin in blood.^15^ Pulse oximetry measures tissue oxygen saturation at two wavelengths (typically 660 and 940 nm), and reliable results are obtained only in the presence of oxyhemoglobin and deoxyhemoglobin. Methemoglobin absorbs light equally at both wavelengths, resulting in an absorbance ratio of one, which corresponds to an oxygen saturation of ∼85%.^19^ However, when the level of methemoglobin is high, the result is unreliable, because the pulse oximeter readings remain stable. ^19^^–^^22^ CO-oximetry is a bench-top analysis method for the evaluation of methemoglobin, and it has been used as the traditional detection tool for methemoglobinemia. Although CO-oximetry can be used to monitor the response to treatment for methemoglobinemia, it is costly and requires an invasive procedure for collecting blood samples from patients. Moreover, the testing of arterial blood gas samples by CO-oximetry requires an order from the physician. Thus the use of CO-oximetry for the measurement of methemoglobin concentrations is limited by the frequency of orders from physicians for a sequential blood gas analysis. A more rapid, simple, and cost-effective noninvasive monitoring method may allow clinicians to perform accurate diagnostic tests for methemoglobinemia.^23^ A pulse CO-oximeter^24^ with a contact probe can measure the percentage of methemoglobin continuously and non-invasively, as well as the percutaneous oxygen saturation. The Masimo Pulse CO-Oximeter (Radical-7, Masimo Corp., Irvine, CA) and The Masimo Pulse oximeter (Radical-5, Masimo Corp., Irvine, CA) have been widely used to measure the percentage of methemoglobin and the percutaneous arterial oxygen saturation (${\mathrm{SpO}}_{2}$), respectively. It has been reported that the Radical-7 had a sensitivity of 97.1% and a specificity of 92.4% for detecting the percentage of methemoglobin ≥10% when the arterial oxygen saturation (${\mathrm{SaO}}_{2}$) is >95%,^25^ whereas the ${\mathrm{SpO}}^{2}$ reading by the Radical-5 showed a sensitivity of 100% and a specificity of 93%.^26^ In terms of the prevention of infectious diseases, it is desirable to monitor methemoglobin and oxygen saturation levels in a non-contact manner. The chemical reactions in the heme group can result in remarkable variations in the hemoglobin absorption spectrum in the visible region. Following the oxidative reaction of hemoglobin, a significant increase in absorption occurs in the red zone of the visible spectrum, and methemoglobin has a characteristic absorption peak at 630  nm.^3^ Changes in the absorption spectrum due to methemoglobin formation are useful for diagnosis of methemoglobinemia and burn wound staging. Some researchers have demonstrated that optical methods have great potential for the non-invasive *in vivo* monitoring of methemoglobin absorption properties; these methods include broadband diffuse optical spectroscopy^27^^–^^30^ and photoacoustic imaging.^31^^,^^32^ Since diffuse reflectance spectroscopy (DRS) can be achieved using simple optical components and apparatuses, it is promising as a non-invasive clinical diagnostic technique. DRS can accurately and simultaneously quantify the *in vivo* concentrations of melanin, oxygenated hemoglobin, and deoxygenated hemoglobin.^33^^–^^35^ Multispectral and hyperspectral imaging techniques can be used to collect spectral information from each pixel in images, which enables the identification of various physiological conditions in living tissues.^36^^–^^38^

In the existing literature, a method has been proposed for the measurements of melanin, oxygenated hemoglobin, and deoxygenated hemoglobin in skin tissues,^35^ and it has been applied to multispectral imaging for visualizing spatiotemporal changes in peripheral hemodynamics and pigmentations in response to physiological stimuli.^39^ In this approach, multiple regression analysis is performed using the absorbance spectrum of human skin between 500 and 600 nm as a response variable and the known extinction coefficient spectra of melanin, oxygenated hemoglobin, and deoxygenated hemoglobin as predictor variables to provide multiple regression coefficients. The concentrations of melanin and hemoglobin are then determined from the regression coefficients using empirical formulas that are deduced numerically in advance. A Monte Carlo simulation (MCS) of light transport in a human skin model is carried out to numerically establish the empirical formulas. We leveraged this approach in our study and extended it to the simultaneous quantification of methemoglobin, oxygenated hemoglobin, and deoxygenated hemoglobin. The purpose of our work was to demonstrate the feasibility of visible spectral diffuse reflectance measurement for *in vivo* monitoring of hemoglobin derivatives and evaluating methemoglobin production and reduction as well as hypoxemia during methemoglobinemia in rats. To confirm the feasibility of the method for quantifying the volume concentrations of methemoglobin, oxygenated hemoglobin, and deoxygenated hemoglobin during methemoglobinemia, we performed *in vivo* animal experiments with rats exposed to sodium nitrite (NaNO_2_) at different doses.

## 2 Materials and Methods

### 2.1 Animal Preparation

Male Wister rats (n=13) weighing 300 to 630 g were used for the animal experiments. All experimental procedures were conducted according to the protocols approved by the Animal Care Committee of Tokyo University of Agriculture and Technology (Approval No. 31-75). Anesthesia of rats was performed with isoflurane and maintained at a depth such that the rat had no response to toe pinching. After the induction of anesthesia, the dorsal region was shaved, and a depilatory agent containing thioglycolic acid was applied to the rat dorsal skin. Thirteen rats were administered a rapid single dose of ${\mathrm{NaNO}}_{2}$ intraperitoneally at the different dose conditions of 25, 37.5, 50, and 75 mg/kg body weight (2-ml dosing volume). Twelve out of thirteen rats were divided into four different dose groups consisting of three rats, respectively, and used only for spectral diffuse reflectance imaging. One out of thirteen rats was used for simultaneous measurements of pulse oximeter signals and spectral diffuse reflectance images.

### 2.2 Experimental Apparatus

**Fig. 2:**
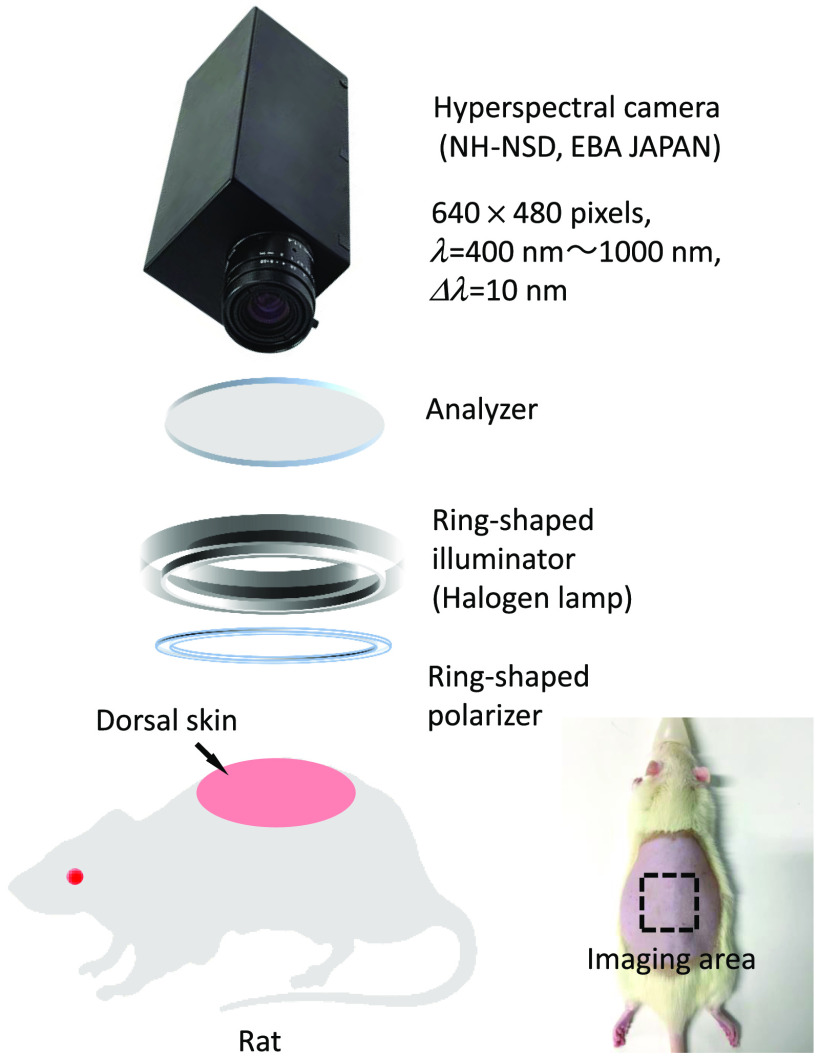
Flow diagram of the process for estimating the concentration of methemoglobin $C_{\text{metHb}},$ oxygenated hemoglobin $C_{\mathrm{HbO}}$, deoxygenated hemoglobin $C_{\text{HbR}}$, and melanin $C_{m}$. (a) Preparation step for establishing the empirical formulas and (b) main process for estimating $C_{\text{metHb}}$, $C_{\text{HbO}}$, $C_{\text{HbR}}$, and $C_{m}$ from the measured diffuse reflectance spectrum.

Figure 2 shows a schematic illustration of the hyperspectral imaging system used in this study. A halogen lamp light source (LA-150SAE, Hayashi Watch Works Co., Ltd., Tokyo, Japan) was used to illuminate the sample surface via a light guide with a ring-shaped illuminator. Diffusely reflected light was received by a hyperspectral camera (NH-NSD, EBA JAPAN, Tokyo, Japan) with a camera lens to acquire a hyperspectral cube. The hyperspectral camera has an internal optical stage-scanning system consisted of a slit, a collimating lens, a transmission diffraction grating, a relay lens, and a two-dimensional charge-coupled device detector sensor. A line in the scene is imaged onto the slit entrance. After collimation, the transmission diffraction grating splits the light into a series of spectral bands that are then focused onto the detector sensor via the relay lens, where one axis (x axis) records the spatial information (along the slit) and the other axis (z axis) records the spectral information. By scanning the internal optical stage along the direction perpendicular to the slit line, the camera collects two-dimensional images for adjacent lines, creating a hyperspectral cube with two spatial dimensions and one spectral dimension; the first two dimensions were spatial (x and y axes) with 640×480  pixels, whereas the third dimension (z axis) was the wavelength, ranging from 400 to 1000 nm at 10-nm intervals. The total acquisition time for one hyperspectral cube was 13 s. The spectral resolution of the camera is 10 nm. A standard white diffuser with 99% reflectance (SRS-99-020, Labsphere Incorporated, North Sutton, NH, USA) was used to correct for the interinstrument differences in the output of the camera and the spatial non-uniformity of the illumination. A ring-shaped polarizer and an analyzer were set in a crossed Nicols alignment to reduce specular reflection from the skin surface. The hyperspectral image data were then stored on a personal computer and analyzed according to the visualizing process described above. The experiments were performed in the darkroom to prevent the effect of ambient light on the spectral imaging. Therefore, ambient light had no significant effect on the imaging. Simultaneously with the optical imaging of skin tissue, the percutaneous arterial oxygen saturation [${\mathrm{SpO}}_{2}$ (%)], heart rate [HR (bpm)], and pulse distention (PD [(μm)] were measured for one rat by a pulse oximeter with a foot sensor (MOUSEOX Pulse Oximeter; Star Life Science, Oakmont, PA, USA). The amplitude of the light absorption signal due to the cardiac pulse, which corresponded to the change in distention of the arterial blood vessels at the sensor location, was taken to be the PD. It can be used as an indicator of local blood flow or peripheral blood volume. After a rest of 5 min, acquisitions of hyperspectral image data were made immediately before the administration of ${\mathrm{NaNO}}_{2}$ (0 min) and at 5, 10, 20, 30, 60, 120, 40, 300, and 360 min after the administration of ${\mathrm{NaNO}}_{2}$, whereas recordings of pulse oximeter signals were made for a total of 360 min at 5-s intervals.

### 2.3 Principle for Quantifying Chromophore Concentrations

#### 2.3.1 Making empirical formulas for quantifying chromophore concentrations

**Fig. 3:**
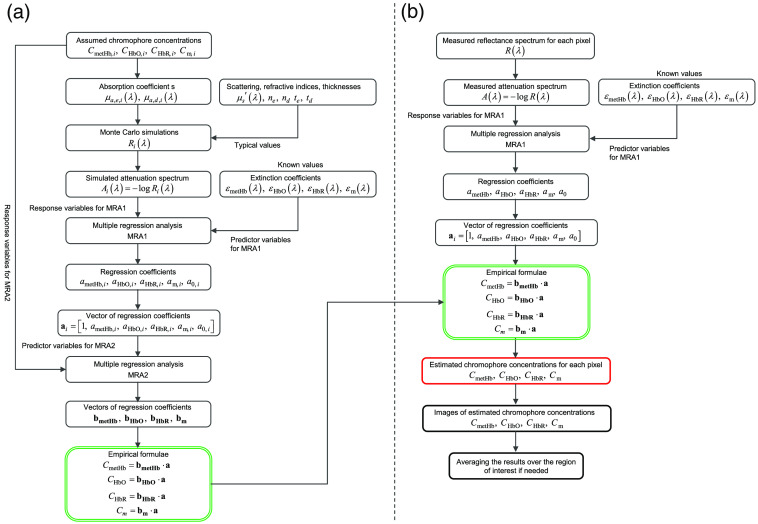
Schematic illustration of the hyperspectral imaging system.

We modified the approach proposed by Nishidate et al.,^35^ which was designed to quantify the three major chromophores of oxygenated hemoglobin, deoxygenated hemoglobin, and melanin. We take methemogobin into account in the skin tissue model in addition to oxygenated hemoglobin, deoxygenated hemoglobin, and melanin. Figure 3(a) shows a flow diagram of establishing the empirical formulas for quantifying concentrations of methemoglobin, oxygenated hemoglobin, deoxygenated hemoglobin, and melanin. The attenuation spectrum A(λ) was defined as

$$A(\lambda )=\log_{10}\frac{1}{R(\lambda )},$$ (1)

where R(λ) is the diffuse reflectance spectrum normalized by the incident light spectrum. Because attenuation due to light scattering can be treated as pseudochromophore,^40^^,^^41^ the attenuation spectrum A(λ) can be approximated as the sum of attenuations due to absorption and scattering in the skin tissue as follows:

$$A(\lambda )=C_{\mathrm{metHb}}l_{d}(\lambda ,C_{\mathrm{metHb}},C_{\mathrm{HbO}},C_{\mathrm{HbR}},\mu_{s}^{\prime })\varepsilon_{\mathrm{metHb}}(\lambda )+C_{\mathrm{HbO}}l_{d}(\lambda ,C_{\mathrm{metHb}},C_{\mathrm{HbO}},C_{\mathrm{HbR}},\mu_{s}^{\prime })\varepsilon_{\mathrm{HbO}}(\lambda )\;+C_{\mathrm{HbR}}l_{d}(\lambda ,C_{\mathrm{metHb}},C_{\mathrm{HbO}},C_{\mathrm{HbR}},\mu_{s}^{\prime })\varepsilon_{\mathrm{HbR}}(\lambda )+C_{m}l_{e}(\lambda ,C_{m},\mu_{s}^{\prime })\varepsilon_{m}(\lambda )+S(\lambda ,\mu_{s}^{\prime }),$$ (2)

where C (M) is the molar concentration, l (cm) is the mean path length, ε (λ) (${\mathrm{cm}}^{-1}\;M^{-1}$) is the molar extinction coefficient, and $S(\lambda ,\mu_{s}^{\prime })$ indicates attenuation due to light scattering in the tissue. The subscripts metHb, HbO, HbR, m, d, and e indicate methemoglobin, oxygenated hemoglobin, deoxygenated hemoglobin, melanin, dermis, and epidermis, respectively. Using A(λ) at λ=550 to 680 nm at 10-nm intervals as the response variable and ε(λ) at the same wavelength range as the predictor variables, the multiple regression analysis can be performed as

$$A(\lambda )=a_{\mathrm{metHb}}\varepsilon_{\mathrm{metHb}}(\lambda )+a_{\mathrm{HbO}}\varepsilon_{\mathrm{HbO}}(\lambda )+a_{\mathrm{HbR}}\varepsilon_{\mathrm{HbR}}(\lambda )+a_{m}\varepsilon_{m}(\lambda )+a_{0},$$ (3)

where $a_{\text{metHb}}$, $a_{\text{HbO}}$, $a_{\text{HbR}}$, $a_{m}$, and $a_{0}$ are the regression coefficients. We refer to this multiple regression analysis as MRA1. In the wavelength range 550 to 680 nm, oxygenated hemoglobin and deoxygenated hemoglobin have isosbestic points at 570 and 585 nm. Oxygenated hemoglobin and methemoglobin have an isosbestic point at 590 nm. Deoxygenated hemoglobin and methemoglobin have isosbestic points around 590 and 660 nm. Reflectance at 560 nm is sensitive to the oxygen state of hemoglobin. Therefore, we chose the wavelength range 550 to 680 nm to use for the multiple regression analysis. As described in Sec. 2.4, we used a hyperspectral camera with a 10-nm spectral resolution in this study. Therefore, we used a spectrum with 10-nm-intervals for this multiple regression analysis. The multiple regression coefficients $a_{\text{metHb}}$, $a_{\text{HbO}}$, $a_{\text{HbR}}$, and $a_{m}$ describe the degree of contribution of $\varepsilon_{\mathrm{metHb}}(\lambda )$, $\varepsilon_{\mathrm{HbO}}(\lambda )$, $\varepsilon_{\mathrm{HbR}}(\lambda )$, and $\varepsilon_{m}(\lambda )$, respectively, to A(λ). Therefore, the values of $a_{\text{metHb}}$, $a_{\text{HbO}}$, $a_{\text{HbR}}$, and $a_{m}$ are related to the products of chromophore concentrations and mean optical path lengths. We used the multiple regression coefficients $a_{\text{metHb}}$, $a_{\text{HbO}}$, $a_{\text{HbR}}$, $a_{m}$, and $a_{0}$ to estimate the concentrations of $C_{\text{metHb}}$, $C_{\text{HbO}}$, $C_{\text{HbR}}$, and $C_{m}$. For this purpose, we assumed the empirical formulas for $C_{\text{metHb}}$, $C_{\text{HbO}}$, $C_{\text{HbR}}$, and $C_{m}$ as

$$C_{\mathrm{metHb}}=b_{\mathrm{metHb}}\cdot a,$$ (4)

$$C_{\mathrm{HbO}}=b_{\mathrm{HbO}}\cdot a,$$ (5)

$$C_{\mathrm{HbR}}=b_{\mathrm{HbR}}\cdot a,$$ (6)

$$C_{m}=b_{m}\cdot a,$$ (7)

where

$$a=[1,a_{\mathrm{metHb}},a_{\mathrm{HbO}},a_{\mathrm{HbR}},a_{m},a_{0}{]}^{T},$$ (8)

$$b_{\mathrm{metHb}}=[b_{\mathrm{metHb},0},b_{\mathrm{metHb},1},b_{\mathrm{metHb},2},b_{\mathrm{metHb},3},b_{\mathrm{metHb},4},b_{\mathrm{metHb},5}],$$ (9)

$$b_{\mathrm{HbO}}=[b_{\mathrm{HbO},0},b_{\mathrm{HbO},1},b_{\mathrm{HbO},2},b_{\mathrm{HbO},3},b_{\mathrm{HbO},4},b_{\mathrm{HbO},5}],$$ (10)

$$b_{\mathrm{HbR}}=[b_{\mathrm{HbR},0},b_{\mathrm{HbR},1},b_{\mathrm{HbR},2},b_{\mathrm{HbR},3},b_{\mathrm{HbR},4},b_{\mathrm{HbR},5}],$$ (11)

$$b_{m}=[b_{m,0},b_{m,1},b_{m,2},b_{m,3},b_{m,4},b_{m,5}].$$ (12)

The symbol $[{]}^{T}$ represents the transposition of a vector. The coefficient vectors $b_{\text{metHb}}$, $b_{\text{HbO}}$, $b_{\text{HbR}}$, and $b_{m}$ are unknown and must be determined before estimating $C_{\text{metHb}}$, $C_{\text{HbO}}$, $C_{\text{HbR}}$, and $C_{m}$. We conducted further multiple regression analyses to establish the vectors $b_{\text{metHb}}$, $b_{\text{HbO}}$, $b_{\text{HbR}}$, and $b_{m}$. In this second multiple regression analysis, $C_{\text{metHb}}$, $C_{\text{HbO}}$, $C_{\text{HbR}}$, and $C_{m}$ were regarded as response variables, and the five regression coefficients ($a_{\text{metHb}}$, $a_{\text{HbO}}$, $a_{\text{HbR}}$, $a_{m}$, and $a_{0}$) that were obtained from MRA1 were regarded as predictor variables to determine the empirical formulas for $C_{\text{metHb}}$, $C_{\text{HbO}}$, $C_{\text{HbR}}$, and $C_{m}$. We refer to this analysis as MRA2.

We used the MCML software developed by Wang et al.^42^ for the MCS to simulate the diffuse reflectance spectra of skin tissues. The simulated spectra were used to derive the datasets of chromophore concentrations and the multiple regression coefficients of $a_{\text{metHb}}$, $a_{\text{HbO}}$, $a_{\text{HbR}}$, $a_{m}$, and $a_{0}$. The simulation model consisted of two layers representing the epidermis and dermis. 5,000,000 photon packets were launched in a single simulation of diffuse reflectance at each wavelength. The absorption coefficients of oxygenated hemoglobin $\mu_{a,\mathrm{HbO}}(\lambda )$ (${\mathrm{cm}}^{-1}$), deoxygenated hemoglobin $\mu_{a,\mathrm{HbR}}(\lambda )$ (${\mathrm{cm}}^{-1}$), methemoglobin $\mu_{a,\mathrm{metHb}}(\lambda )$ (${\mathrm{cm}}^{-1}$), and melanin $\mu_{a,m}(\lambda )$ (${\mathrm{cm}}^{-1}$) were obtained from the values of $\varepsilon_{\mathrm{HbO}}(\lambda )$ (${\mathrm{cm}}^{-1}\;M^{-1}$), $\varepsilon_{\mathrm{HbR}}(\lambda )$ (${\mathrm{cm}}^{-1}\;M^{-1}$), $\varepsilon_{\mathrm{metHb}}(\lambda )$ (${\mathrm{cm}}^{-1}\;M^{-1}$), and $\varepsilon_{\mathrm{mel}}(\lambda )$ (${\mathrm{cm}}^{-1}\;M^{-1}$) as shown in Fig. 1. The value of $\mu_{a}$ (λ) (${\mathrm{cm}}^{-1}$) was derived from the product of the molar concentration C (M) and the molar extinction coefficient ε(λ) (${\mathrm{cm}}^{-1}\;M^{-1}$) as, $\mu_{a}$ (λ)=2.303Cε(λ). The absorption coefficients $\mu_{a,m}(\lambda )$ for 10 different melanin concentrations were input for the epidermis layer in the MCS as $C_{m}=1$ to 10 vol. % at 1-vol. % intervals. We assumed that the whole blood with 2.32 mM of hemoglobin is 100% volume concentration of total hemoglobin ($C_{\text{HbT}}=100$ vol. %). The sum of the absorption coefficients of oxygenated hemoglobin $\mu_{a,\mathrm{HbO}}(\lambda )$, deoxygenated hemoglobin $\mu_{a,\mathrm{HbR}}(\lambda )$, and methemoglobin $\mu_{a,\mathrm{metHb}}(\lambda )$ represents the absorption coefficients of total hemoglobin $\mu_{a,\mathrm{HbT}}(\lambda )$ (${\mathrm{cm}}^{-1}$). The absorption coefficients for total hemoglobin $\mu_{a,\mathrm{HbT}}(\lambda )$ for the values of $C_{\text{HbT}}=0.2$ to 1.0 vol. % at 0.2-vol. % intervals were input for the dermis layer in the MCS. Tissue oxygen saturation (${\mathrm{StO}}_{2}$) and methemoglobin saturation (StMet) were determined by $\mu_{a,\mathrm{HbO}}(\lambda )/\mu_{a,\mathrm{HbT}}(\lambda )$ and $\mu_{a,\mathrm{metHb}}(\lambda )/\mu_{a,\mathrm{HbT}}(\lambda )$, respectively, and values ranged from 0% to 100% were used for the simulation. The refractive index of the epidermis and dermis layers was assumed to be same and fixed at 1.4. The thicknesses of epidermis and dermis layers were set to 0.06 and 4.94 mm, respectively. The reduced scattering coefficient $\mu_{s}^{\prime }(\lambda )({\mathrm{cm}}^{-1})$ calculated from the typical values for the scattering coefficient^35^ $\mu_{s}(\lambda )$ (${\mathrm{cm}}^{-1}$) and anisotropy factor^35^ g(λ) were used for both epidermis and dermis layers. The value of $\mu_{s}^{\prime }$ at 550 nm was $31.62\;{\mathrm{cm}}^{-1}$. In total, 1550 diffuse reflectance spectra at λ=550 to 680 nm at 10-nm intervals were simulated under the various combinations of $C_{\text{metHb}}$ vol. %, $C_{\text{HbO}}$ vol. %, $C_{\text{HbR}}$ vol. %, and $C_{m}$ vol. %. The MRA1 analysis for each simulated spectrum generated 1550 sets of vector a and concentrations of $C_{\text{metHb}}$ vol. %, $C_{\text{HbO}}$ vol. %, $C_{\text{HbR}}$ vol. %, and $C_{m}$ vol. %. The coefficient vectors $b_{\text{metHb}}$, $b_{\text{HbO}}$, $b_{\text{HbR}}$, and $b_{m}$ were determined statistically by performing MRA2. In this way, we used both multiple regression analysis and the MCS together to establish the empirical model.

#### 2.3.2 Estimating chromophore concentrations using the established empirical formulas

Figure 3(b) shows the main process for estimating concentrations of methemoglobin, oxygenated hemoglobin, deoxygenated hemoglobin, and melanin from the measured diffuse reflectance spectrum using the established empirical formulas. The measured diffuse reflectance spectrum R(λ) is converted into the attenuation spectrum A(λ). MRA1 is then performed using the measured A(λ) at λ=550 to 680 nm at 10-nm intervals as the response variable and ε(λ) at the same wavelength range as the predictor variables. The resultant multiple regression coefficients $a_{\text{metHb}}$, $a_{\text{HbO}}$, $a_{\text{HbR}}$, $a_{m}$, and $a_{0}$ are substituted into the empirical formulas established in the preparation step described in Sec 2.3.1 and finally the estimated concentrations of methemoglobin $C_{\text{metHb}}$ vol. %, oxygenated hemoglobin $C_{\text{HbO}}$ vol. %, deoxygenated hemoglobin $C_{\text{HbR}}$ vol. %, and melanin $C_{m}$ vol. % are obtained. The above procedure was done for each pixel.

### 2.4 In Silico Experiments

We performed *in silico* experiments with diffuse reflectance samples generated by the MCS to validate the accuracy of the proposed method. For test samples, the values of $C_{\text{HbT}}$ were set to 0.3, 0.5, and 0.7 vol. %, whereas those of $C_{m}$ were set to 1.5, 3.5, and 5.5 vol. % The values of ${\mathrm{StO}}_{2}$ were ranged from 2% to 72%, whereas those of StMet were set to 20%, 50%, and 80%. Other conditions for the MCS were fixed to be the same as those in Sec. 2.3.1. In total, 81 diffuse reflectance spectra at λ=550 to 680 nm at 10-nm intervals were generated under the combinations of $C_{\text{HbT}}$, $C_{m}$, ${\mathrm{StO}}_{2}$, and StMet.

### 2.5 Statistical Analysis

To assess the results from *in silico* experiments, Pearson’s correlation coefficient and root-mean-square error (RMSE) were calculated using the ground truth values and estimated values by the proposed method. A probability value of p<0.05 indicates statistical significance. We used only one area of each rat for data analysis of hyperspectral images. Region of interest (ROI) of 300×300  pixels was set in each image and the mean and the standard deviation (SD) over the ROI were calculated for the analysis of time courses in $C_{\text{metHb}}$ $C_{\text{HbO}}$, $C_{\text{HbR}}$, $C_{\text{HbT}}$ $C_{m}$, ${\mathrm{StO}}_{2}$, and StMet. Therefore, data are expressed as mean ± SD. In order to compare the difference in each parameter across the dose conditions, the mean over the three rats in each group was also calculated.

## 3 Results and Discussion

**Fig. 4:**
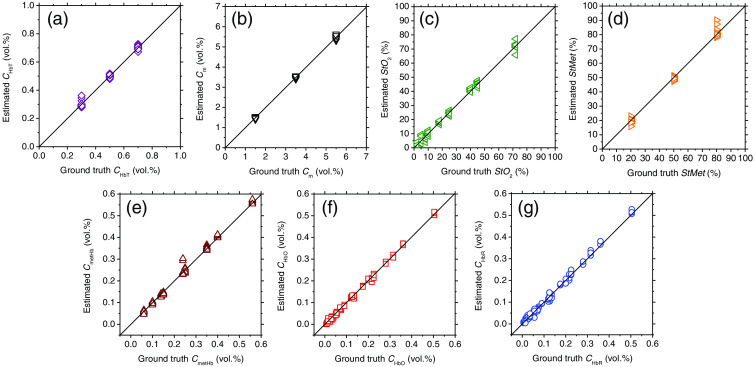
Comparisons between the estimated and ground truth values for (a) total hemoglobin concentration $C_{\text{HbT}}$; (b) melanin concentration $C_{m}$; (c) tissue oxygen saturation ${\mathrm{StO}}_{2}$; (d) methemoglobin saturation StMet; (e) methemoglobin concentration $C_{\text{metHb}}$; (f) oxygenated hemoglobin concentration $C_{\mathrm{HbO}}$; and (g) deoxygenated hemoglobin concentration $C_{\mathrm{HbR}}$, obtained from the *in silico* experiments.

We first validated the accuracy of the proposed method by *in silico* experiments with diffuse reflectance samples generated by the MCS (Fig. 4). The estimated and ground truth values for total hemoglobin concentration $C_{\text{HbT}}$, melanin concentration $C_{m}$, tissue oxygen saturation ${\mathrm{StO}}_{2}$, methemoglobin saturation StMet, methemoglobin concentration $C_{\text{metHb}}$, oxygenated hemoglobin concentration $C_{\mathrm{HbO}}$, and deoxygenated hemoglobin concentration $C_{\text{HbR}}$ are shown in Figs. 4(a)–(g), respectively. The estimated values are well correlated with the ground truth values for given ranges of each parameter. The value of Pearson’s correlation coefficient between the estimated and ground truth values of $C_{\text{HbT}}$, $C_{m}$, ${\mathrm{StO}}_{2}$, StMet, $C_{\text{metHb}}$, $C_{\text{HbO}}$, and $C_{\text{HbR}}$ were calculated to be 0.997 (p<0.0001), 0.999 (p<0.0001), 0.997 (p<0.0001), 0.998 (p<0.0001), 0.996 (p<0.0001), 0.998 (p<0.0001), and 0.997 (p<0.0001), respectively. The RMSEs of $C_{\text{HbT}}$, $C_{m}$, ${\mathrm{StO}}_{2}$, StMet, $C_{\text{metHb}}$, $C_{\text{HbO}}$, and $C_{\text{HbR}}$ were 0.39%, 0.18%, 3.06%, 0.45%, 0.70%, 3.03%, and 2.21%, respectively.

**Fig. 5:**
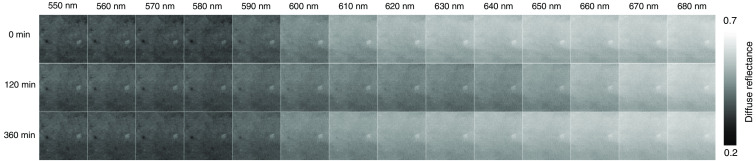
Typical spectral diffuse reflectance images of rat dorsal skin measured by the hyperspectral camera at 0, 120, and 360 min after the administration of ${\mathrm{NaNO}}_{2}$. Three representative time points are illustrated to show the largest differences, i.e., at 120 min the diffuse reflectance at 610 to 650 nm was the lowest.

**Fig. 6:**
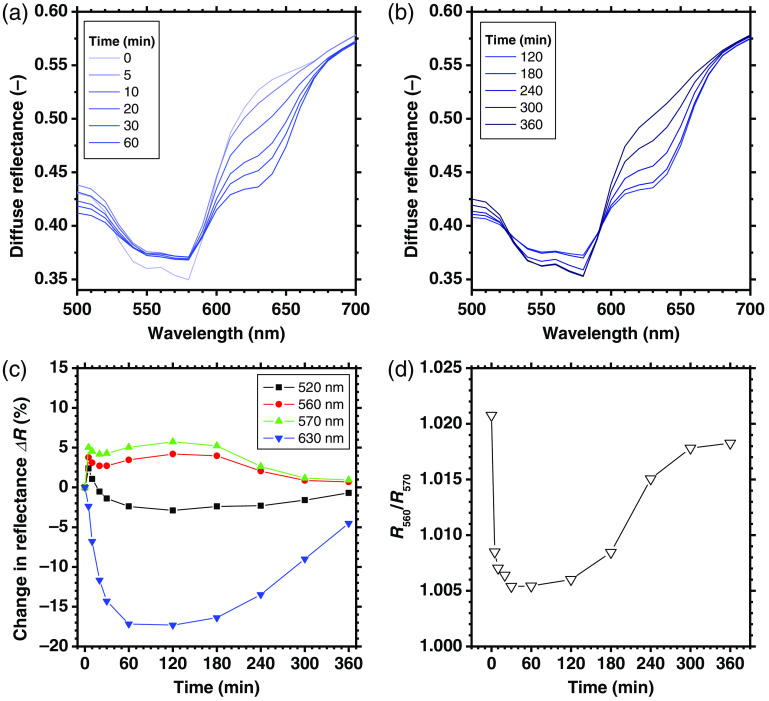
Typical time course of diffuse reflectance spectra obtained from rat dorsal skin before and after the administration of ${\mathrm{NaNO}}_{2}$ for (a) 0 to 60 min and (b) 120 to 360 min. Each spectrum is the average value over the ROI in the spectral diffuse reflectance image measured by the hyper spectral camera. (c) The percent change in diffuse reflectance relative to the value at 0 min for 520, 560, 570, and 630 nm. (d) Time course of the ratio of diffuse reflectance at 560 nm and that at 570 nm.

We examined the spectral diffuse reflectance images obtained from rat dorsal skin at 0, 120, and 360 min after the administration of ${\mathrm{NaNO}}_{2}$ to confirm the impact of light absorption by methemoglobin on the spectrum. As expected from the absorption spectrum of methemoglobin shown in Fig. 1, the diffuse reflectance values in the range from 610 to 650 nm were clearly decreased at 120 min after the administration of ${\mathrm{NaNO}}_{2}$ (Fig. 5). We found that an increase in metHb caused by ${\mathrm{NaNO}}_{2}$ administration would lead to a maximum percentage decrease of 17% in diffuse reflectance at 630 nm [Fig. 6 (c)]. The diffuse reflectance at 520 nm showed that there was an initial slight increase of 2% at 5 min, followed by a decrease of 5% with a minimum value at 120 min, then it returned to its normal level at 360 min [Fig. 6 (c)]. A decrease in $R_{560}/R_{570}$, i.e., the local peak of the diffuse reflectance at 560 nm relative to that at 570 nm at 10 to 60 min after ${\mathrm{NaNO}}_{2}$ administration reflected the deoxygenation of hemoglobin [Fig. 6 (d)]. Methemoglobin, oxygenated hemoglobin, and deoxygenated hemoglobin have almost the same molar extinction coefficient at 520 nm, as shown in Fig. 1. Therefore, the change in diffuse reflectance at 520 nm implied a temporary increase in the volume concentration of total hemoglobin.

**Fig. 7:**
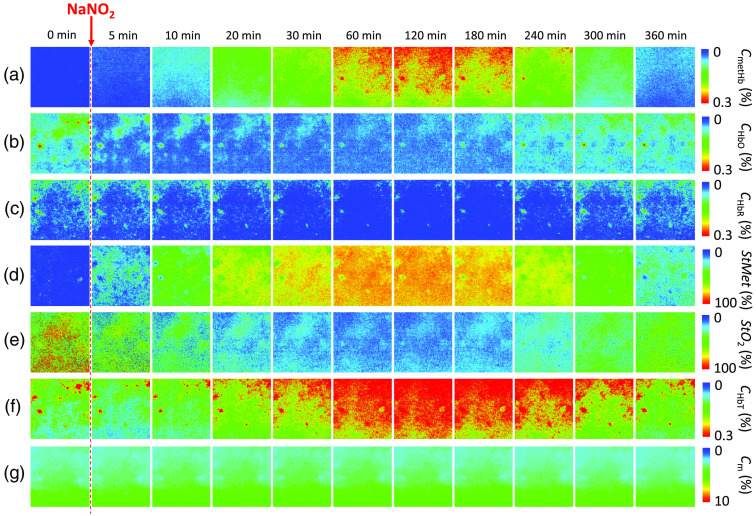
Typical sequential images of (a) methemoglobin concentration $C_{\text{metHb}}$; (b) oxygenated hemoglobin concentration $C_{\text{HbO}}$; (c) deoxygenated hemoglobin concentration $C_{\text{HbR}}$; (d) methemoglobin saturation StMet; (e) tissue oxygen saturation ${\mathrm{StO}}_{2}$; (f) total hemoglobin concentration $C_{\text{HbT}}$; and (g) melanin concentration $C_{m}$ obtained from rat dorsal skin before and after the administration of ${\mathrm{NaNO}}_{2}$ at a dose of 50 mg/kg. Data were measured by the proposed method.

**Fig. 8:**
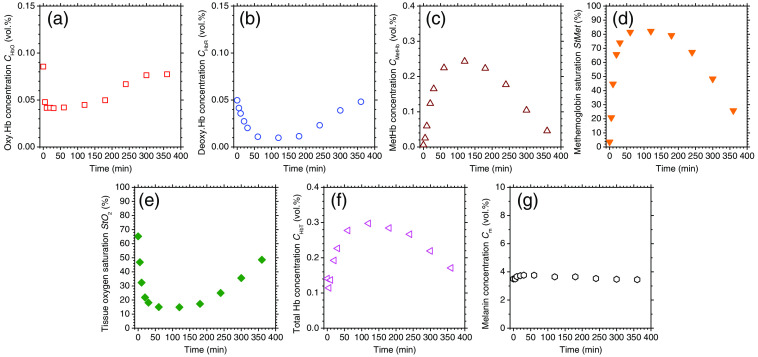
Typical time courses of (a) methemoglobin concentration $C_{\text{metHb}}$; (b) oxygenated hemoglobin concentration $C_{\text{HbO}}$; (c) deoxygenated hemoglobin concentration $C_{\text{HbR}}$; (d) methemoglobin saturation StMet; (e) tissue oxygen saturation ${\mathrm{StO}}_{2}$; (f) total hemoglobin concentration $C_{\mathrm{HbT}}$; and (g) melanin concentration $C_{m}$ averaged over the entire region of each corresponding image shown in Fig. 7.

We applied the proposed method to the spectral diffuse reflectance images obtained from rat dorsal skin before and after the administration of ${\mathrm{NaNO}}_{2}$ at a dose of 50 mg/kg to evaluate quantitatively changes in chromophore concentrations. Figure 7 shows typical sequential images of (a) $C_{\text{metHb}}$, (b) $C_{\text{HbO}}$, (c) $C_{\text{HbR}}$, (d) StMet, (e) ${\mathrm{StO}}_{2}$, (f) $C_{\text{HbT}}$, and (g) $C_{m}$ obtained from rat dorsal skin before and after the administration of ${\mathrm{NaNO}}_{2}$ at a dose of 50 mg/kg. Figure 8 shows the typical time courses of (a) $C_{\text{metHb}}$, (b) $C_{\text{HbO}}$, (c) $C_{\text{HbR}}$, (d) StMet, (e) ${\mathrm{StO}}_{2}$, (f) $C_{\text{HbT}}$, and (g) $C_{m}$ averaged over the entire region of each corresponding image shown in Fig. 7. Both $C_{\text{metHb}}$ and StMet increased after the administration of ${\mathrm{NaNO}}_{2}$, whereas $C_{\text{HbO}}$ and $C_{\text{HbR}}$ decreased and increased, respectively, which was indicative of temporary methemoglobinemia. At 360 min after the administration of ${\mathrm{NaNO}}_{2}$, both $C_{\text{metHb}}$ and StMet returned to their normal levels due to the self-reductive systems of the rat. The value of ${\mathrm{StO}}_{2}$ decreased after the administration of ${\mathrm{NaNO}}_{2}$, indicating temporary hypoxemia caused by methemoglobinemia as expected (Figs. 7 and 8). The value of $C_{\text{HbT}}$ showed an initial decrease of 0.03 vol. %, followed by a profound increase of 0.16 vol. % after the administration of ${\mathrm{NaNO}}_{2}$. The first phase of total hemoglobin change coincided with the decrease in both $C_{\text{HbO}}$ and $C_{\text{HbR}}$ (Fig. 8).

**Fig. 9:**
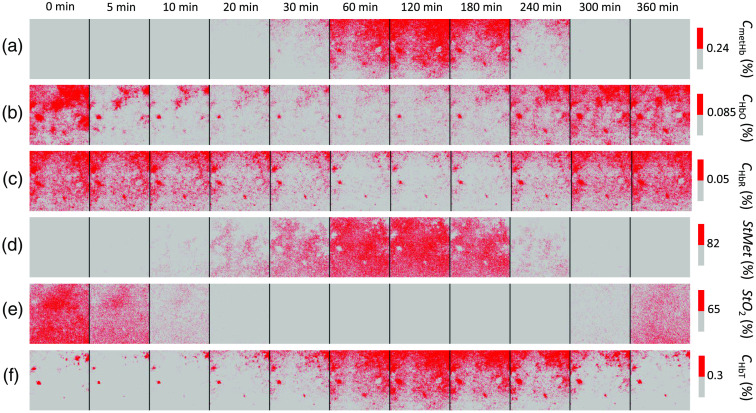
Segmentation results for sequential images shown in Fig. 7. (a) methemoglobin concentration $C_{\text{metHb}}$; (b) oxygenated hemoglobin concentration $C_{\text{HbO}}$; (c) deoxygenated hemoglobin concentration $C_{\text{HbR}}$; (d) methemoglobin saturation StMet; (e) tissue oxygen saturation ${\mathrm{StO}}_{2}$; and (f) total hemoglobin concentration $C_{\text{HbT}}$. Red color and gray color show a pixel above and below a threshold, respectively. The average values overall pixels of images at 0 min were selected as the threshold pixel values for $C_{\text{HbO}}$, $C_{\text{HbR}}$, and ${\mathrm{StO}}_{2}$, whereas those at 120 min were selected as the threshold pixel values for $C_{\text{metHb}}$, $C_{\text{HbT}}$, and StMet. The threshold pixel values for $C_{\text{metHb}}$, $C_{\text{HbO}}$, $C_{\text{HbR}}$, StMet, ${\mathrm{StO}}_{2}$, and $C_{\text{HbT}}$ were 0.24 vol. %, 0.085 vol. %, 0.05 vol. %, 82%, 65%, and 0.3 vol. %, respectively.

To confirm the applicability of the method to imaging applications in monitoring of hemoglobin derivatives, we investigated variations in spatial distributions of $C_{\text{metHb}}$, $C_{\text{HbO}}$, $C_{\text{HbR}}$, StMet, ${\mathrm{StO}}_{2}$, and $C_{\text{HbT}}$. Figure 9 shows segmentation results for sequential images shown in Fig. 7. We selected a threshold pixel value for each chromophore image, and segmented all pixels above the threshold value, and looked at how the segmented regions change over time. The average values overall pixels of images at 0 min after the administration of ${\mathrm{NaNO}}_{2}$ were selected as the threshold pixel values for $C_{\text{HbO}}$, $C_{\text{HbR}}$, and ${\mathrm{StO}}_{2}$, whereas those at 120 min after the administration of ${\mathrm{NaNO}}_{2}$ were selected as the threshold pixel values for $C_{\text{metHb}}$, $C_{\text{HbT}}$, and StMet. The threshold pixel values for $C_{\text{metHb}}$, $C_{\text{HbO}}$, $C_{\text{HbR}}$, StMet, ${\mathrm{StO}}_{2}$, and $C_{\text{HbT}}$ were 0.24 vol. %, 0.085 vol. %, 0.05 vol. %, 82%, 65%, and 0.3 vol. %, respectively. The region of segmented pixels for $C_{\text{HbT}}$ began to increase in the upper half of the image at 30 min after the administration of ${\mathrm{NaNO}}_{2}$ and spreads to the lower half of the image until 120 min after the administration of ${\mathrm{NaNO}}_{2}$ [Fig. 9(f)]. The same tendency can be observed in the segmented image of $C_{\text{metHb}}$ [Fig. 9(a)]. On the other hand, the decreases in the regions of segmented pixels for both $C_{\text{HbO}}$ and $C_{\text{HbR}}$ spread from the upper side to the lower side of the image until 120 min after the administration of ${\mathrm{NaNO}}_{2}$ [Figs. 9(b) and 9(c)]. The cause of the variations in spatial distribution of $C_{\text{metHb}}$, $C_{\text{HbO}}$, $C_{\text{HbR}}$, and $C_{\text{HbT}}$ is unclear. One possible explanation for those variations is heterogeneous microvascular perfusion distribution. Changes in the regions of segmented pixels for StMet are spatially uniform, which is probably due to that StMet is calculated as the ratio of methemoglobin to total hemoglobin [Fig. 9(d)]. The same applies to the segmentation image of ${\mathrm{StO}}_{2}$ that is the ratio of oxygenated hemoglobin to total hemoglobin [Fig. 9(e)].

**Fig. 10:**
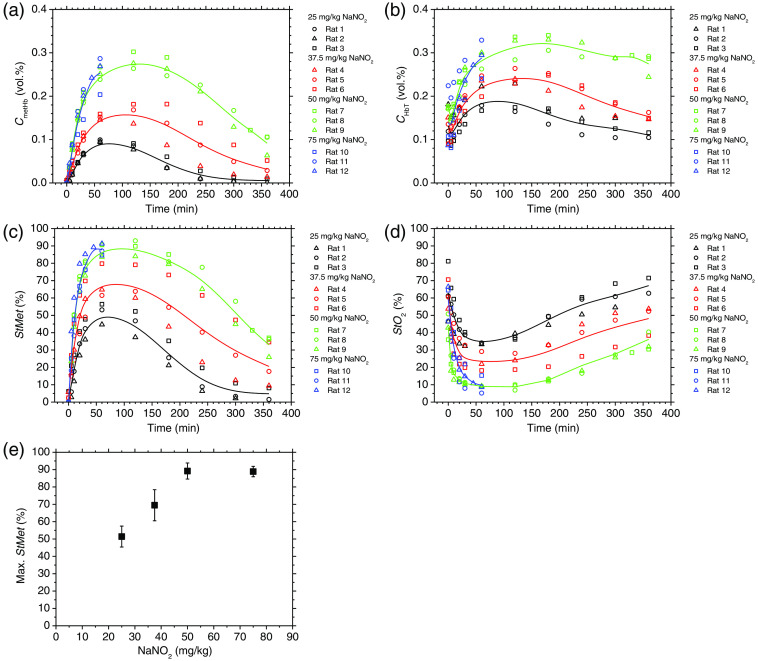
Time courses of (a) methemoglobin concentration $C_{\text{metHb}}$; (b) total hemoglobin concentration $C_{\text{HbT}}$; (c) methemoglobin saturation StMet; and (d) tissue oxygen saturation ${\mathrm{StO}}_{2}$ for various doses of ${\mathrm{NaNO}}_{2}$. (e) Relationship between the maximum value of StMet and the ${\mathrm{NaNO}}_{2}$ dose. Data were obtained by the proposed method. Solid lines in (a)–(d) are means of three rats for each dose group. Plots and error bars in (e) show means and SDs of three rats for each dose group.

We compared the time courses of (a) $C_{\text{metHb}}$, (b) $C_{\text{HbT}}$, (c) StMet, and (d) ${\mathrm{StO}}_{2}$ for ${\mathrm{NaNO}}_{2}$ doses of 25, 37.5, 50, and 75 mg/kg to investigate the dose-dependent effects of ${\mathrm{NaNO}}_{2}$ (Fig. 10). The values of $C_{\text{metHb}}$ and StMet rapidly increased with a half-maximum time of <20  min, and the time required to return to the normal levels increased proportionally with the dose [Figs. 10(a) and 10(c)]. On the other hand, at each dose condition, the time course of ${\mathrm{StO}}_{2}$ dramatically dropped at 60 min after the administration of ${\mathrm{NaNO}}_{2}$, and then gradually increased again [Fig. 10(d)]. The minimum averaged values of ${\mathrm{StO}}_{2}$ during methemoglobinemia were 33.7%±0.4%, 23.1%±5.6%, 8.8%±1.7%, and 9.7%±5.1% on average for ${\mathrm{NaNO}}_{2}$ doses of 25, 37.5, 50, and 75 mg/kg, respectively. The values of ${\mathrm{StO}}_{2}$ were negatively correlated to those of $C_{\text{metHb}}$ and StMet. All three rats with the dose condition of 75 mg/kg body weight died at 60 to 69 min after the administration of ${\mathrm{NaNO}}_{2}$, whereas those with the other dose conditions survived. Therefore, the median lethal dose was speculated to be within the dose of 50 to 75 mg/kg. We found that the maximum value of StMet increased with increasing dose up to 50 mg/kg, in which it reached a plateau [Fig. 10 (e)].

**Fig. 11:**
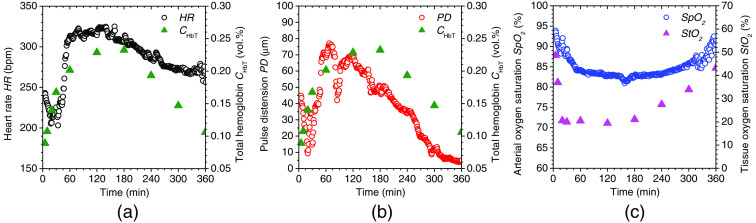
Time courses of (a) HR and total hemoglobin concentration $C_{\mathrm{HbT}}$; (b) PD and total hemoglobin concentration $C_{\mathrm{HbT}}$; and (c) percutaneous arterial oxygen saturation ${\mathrm{SpO}}_{2}$ and tissue oxygen saturation ${\mathrm{StO}}_{2}$ for rat after the administration of ${\mathrm{NaNO}}_{2}$ at a dose of 37.5 mg/kg. HR, PD, and ${\mathrm{SpO}}_{2}$ were measured by the commercial existing pulse oximeter, whereas $C_{\mathrm{HbT}}$ and ${\mathrm{StO}}_{2}$ were obtained by the proposed method.

In order to discuss the results of total hemoglobin concentration $C_{\text{HbT}}$ after the administration of ${\mathrm{NaNO}}_{2}$, we compared the time course of $C_{\text{HbT}}$ obtained by the proposed method with HR and pulse distension (PD) measured by the commercial existing pulse oximeter [Figs. 11(a) and 11(b)]. We found that the HR significantly increased to a maximum of 323 bpm at 135 min after the administration of ${\mathrm{NaNO}}_{2}$, then it gradually decreased for 210 min [Fig. 11(a)]. This increase in HR corresponded to tachycardia, which is a symptom of methemoglobinemia.^43^ The PD after the administration of ${\mathrm{NaNO}}_{2}$ initially decreased to 9.3  μm, but then it significantly increased to a maximum of 76.2  μm at 63 min, then subsequently returned to normal levels [Fig. 11(b)]. The profound increase in PD indicated an elevated cardiac output caused by tachycardia and the resultant increase in peripheral blood volume to compensate for the hypoxia and hypoxemia during methemoglobinemia; this was in agreement with the time course of $C_{\text{HbT}}$ obtained by the proposed method.

We also compared the time course of ${\mathrm{StO}}_{2}$ obtained by the proposed method with percutaneous arterial oxygen saturation ${\mathrm{SpO}}_{2}$ measured by the commercial existing pulse oximeter. We found that the ${\mathrm{SpO}}_{2}$ significantly dropped after the administration of ${\mathrm{NaNO}}_{2}$, then remained around 82.5% for 160 min [Fig. 11(c)]. This observation was in agreement with several reports of ${\mathrm{SpO}}_{2}$ measured by conventional pulse oximetry during methemoglobinemia.^19^^–^^22^ It has been reported that methemoglobinemia can cause a discrepancy between the values of ${\mathrm{SpO}}_{2}$ measured by a conventional pulse oximeter and the actual arterial oxygen saturation (${\mathrm{SaO}}_{2}$) calculated by arterial blood gas measurement.^19^^–^^22^ The values of ${\mathrm{SpO}}_{2}$ are not proportional to the actual ${\mathrm{SaO}}_{2}$ during methemoglobinemia, since elevated methemoglobin levels (up to 60%) force the ${\mathrm{SpO}}_{2}$ value to be around 85%, whereas ${\mathrm{SaO}}_{2}$ reaches ∼35%.^19^ When methemoglobin is elevated, the ${\mathrm{SpO}}_{2}$ values measured by conventional pulse oximetry are inaccurate and can falsely diagnose the actual status of patients.

In contrast, the values of ${\mathrm{StO}}_{2}$ obtained by the proposed method were dramatically decreased with a minimum of 19.9% as shown in Fig. 11(c). Since the value of ${\mathrm{StO}}_{2}$ estimated by the proposed method represents the oxygen saturation for a mixture of ${\mathrm{SaO}}_{2}$ and ${\mathrm{SvO}}_{2}$, it should be calculated to be lower than the ${\mathrm{SaO}}_{2}$ value. Considering that the ${\mathrm{SaO}}_{2}$ value has been reported to be ∼35%during methemoglobinemia,^19^ it is reasonable that the minimum averaged values of ${\mathrm{StO}}_{2}$ during methemoglobinemia showed 33.7%±0.4%, 23.1%±5.6%, 8.8%±1.7%, and 9.7%±5.1% on average for ${\mathrm{NaNO}}_{2}$ doses of 25, 37.5, 50, and 75 mg/kg, respectively, as shown in Fig. 10(d). The results for ${\mathrm{StO}}_{2}$ indicated that the proposed method can be used to evaluate hypoxemia during methemoglobinemia.

The advantage of the diffuse reflectance imaging over the DRS is the ability to visualize the spatial distribution of chromophores. For example, the spatial distributions of oxygenated hemoglobin concentration, deoxygenated hemoglobin concentration, methemoglobin concentration, and tissue oxygen saturation are important for evaluating the depth of burn injury to classify the severity of burn injury. This issue should be studied further in the future. Near-infrared spectroscopy measurements^29^^–^^32^ also considered lipids and water as major chromophores in addition to hemoglobin derivatives because lipids and water have distinct absorption peaks in the near-infrared wavelength region. On the other hand, there is no significant absorption by lipids or water in the wavelength range from 550 to 680 nm we used. Therefore, we did not consider lipids and water as major chromophores in this study. The current empirical formulas for chromophores were derived from the MCS with a typical reduced scattering coefficient spectrum. However, the reduced scattering coefficient spectrum usually differs among body parts and may vary with the age of the subjects. Correct estimation of the scattering properties or consideration of the variations in the reduced scattering coefficient spectrum is essential to precisely estimate the chromophore concentrations. We assumed the typical thicknesses of epidermis and the dermis in the MCS to establish the empirical model. The assumed epidermis and dermis thicknesses could have an impact to the results. The probability that light is absorbed by melanin in the epidermis will be higher than the probability that light is absorbed by hemoglobin in the dermis as epidermis becomes thicker. Therefore, if the thickness of epidermis is larger than the typical value, melanin concentration in epidermis or hemoglobin derivatives concentrations may be under- or overestimated, respectively. The full thickness of the skin tissue model was set to 5 mm in the MCS. The simulated diffuse reflectance could be increased if the total thickness of skin tissue model is larger than 5 mm. Hence, the MCS on a thicker tissue model with a semi-infinite geometry could change the results. However, the possibility of relative measurements of oxygenated hemoglobin, deoxygenated hemoglobin, methemoglobin, and melanin in the skin tissue could still prove valuable. Although we did not consider the polarized photons in the MCS, the diffuse reflectance was calculated separately from the specular reflectance in the simulation and used for in a data set for the empirical model. The quantification accuracy may be improved by taking into account the polarization effects in the MCS. We performed the MCS using the MCML software. The MCML software basically output the physical quantities of photon absorption, fluence, diffuse reflectance, specular reflectance, and total transmittance. It could be possible to estimate the mean path length if we modified the MCML source code. The estimated mean path length should be compared with the values of a and b in the future. The present method lacks depth resolution, because it relies on the integration of all diffusing reflection information along the depth direction. Moreover, the present method is based on the MCS model, which assumes uniformly distributed scattering and absorption properties. The current algorithm performs the multiple regression analysis using each pixel in the spectral image to calculate the multiple regression coefficients. This process is relatively time-consuming, and the computational load increases when the number of pixels in an image for analysis increases. Therefore, this method may not be suitable for real-time high temporal and spatial resolution imaging of chromophores *in vivo*. Although hyperspectral imaging techniques have been widely used for scientific purposes in the laboratory environment, it is impractical for clinical use due to the large size and high cost of commercial setups. It is advantageous that the proposed algorithm can also be combined with the RGB camera-based spectral image reconstruction technique based on the Wiener estimation method.^44^ This is promising for the establishment of a compact and affordable imaging system that can simultaneously evaluate the spatiotemporal distributions of methemoglobin, oxygenated hemoglobin, and deoxygenated hemoglobin in clinical point-of-care testing. Such a device would have an enormous impact on the health of people of all ages worldwide, and this issue should be studied further in the future.

## 4 Conclusions

In summary, this study demonstrated the usefulness of a new non-contact measuring method based on DRS for the simultaneous evaluations of the precutaneous volume concentrations of methemoglobin, oxygenated hemoglobin, deoxygenated hemoglobin, and melanin as well as tissue oxygen saturation and methemoglobin saturation. *In vivo* experiments with rat dorsal skin before and after the administration of ${\mathrm{NaNO}}_{2}$ at doses of 25, 37.5, 50, and 75 mg/kg were performed to induce methemoglobinemia. Both the methemoglobin concentration and methemoglobin saturation rapidly increased with a half-maximum time of <20  min. They reached their maximal values nearly 60 min after the administration of ${\mathrm{NaNO}}_{2}$, and the time required to return to the normal levels increased proportionally with the dose, which implied that the methemoglobinemia was temporary. The values of tissue oxygen saturation dramatically dropped after the administration of ${\mathrm{NaNO}}_{2}$, and then they gradually increased for each dose condition, indicating temporary hypoxemia caused by methemoglobinemia. The time course of the peripheral volume concentration of total hemoglobin during methemoglobinemia obtained by the proposed method reflects both the HR and PD that are measured by the commercially available pulse oximeter. The results of this study showed that the proposed method has potential for the simultaneous evaluations of the methemoglobin level, regional peripheral blood volume, and hypoxemia during methemoglobinemia.
